# A conceivable mechanism of harm in a stretched “teen lung”

**DOI:** 10.1186/s13054-018-2284-6

**Published:** 2019-01-10

**Authors:** João Batista Borges

**Affiliations:** Centre for Human and Applied Physiological Sciences, King’s College, London, UK

**Keywords:** Acute respiratory distress syndrome, Mechanical ventilation, Ventilator-induced lung injury

Atelectrauma vs volutrauma is indeed a misleading dilemma [1]. We recently reported evidence that normally and poorly aerated units were the paramount hotspots of the inflammatory process related to ventilator-induced lung injury (VILI), even during 27-h application of the current standard of care for lung-protective ventilation [2]. The aerated, ventilated, and “apparently” spared units were the ones that suffered the most damaging effects. This may be attributed to the small-aerated lung (the so-called “baby lung”), which receives most ventilation and therefore is exposed to nonphysiologic stretch. The more dependent and more nondependent regions may redirect the harm to the normally and poorly aerated units by diverting the tidal volume to those regions, submitting them to higher tidal stretch. The local consequences in more nondependent and dependent units may be less important than the cyclic stretches driven to the baby lung.

On a much smaller spatial scale, it has been demonstrated by utilizing synchrotron-based X-ray tomographic microscopy that local strains are up to four times higher than the global strain, and that the strain hotspots occurred within the thinnest parts, where there is less tissue to resist the deformation [3]. This likely leads to an uneven strain distribution throughout the parenchymal tissue, with thin regions becoming overstretched, whereas regions with tissue accumulation remain unchallenged.

High mean airway pressures without optimum lung recruitment, i.e., on top of a partly/suboptimally recruited lung, may potentially exacerbate these local stretches [4]. We may interpret the OSCILLATE [4] trial results as the outcome of a conceivable mechanism of harm in a stretched and inhomogeneous “teen lung”. Its data point out a possible mechanism of injury associated with submitting suboptimally recruited lungs (“teen lungs”) to high airway pressures. The maximal PaO_2_/F_I_O_2_ achieved by the OSCILLATE “open lung” strategy was ≤ 160 mmHg, but values around 250 mmHg correspond still to 28% parenchymal mass collapse on computed tomography [5]. Lung-dependent sticky atelectasis usually requires pressures > 40 cmH_2_O to recruit [5]. An insufficient, not individualized and/or delayed lung recruitment strategy—followed or not by an appropriate positive end-expiratory pressure titration—can give rise to a “heterogeneously stretched teen lung”, prone to relevant mechanisms of injury, especially when high pressures are applied. It follows that the search for improvements in lung homogeneity and acute respiratory distress syndrome patients’ outcomes by means of an optimum (instead of partial) and comprehensive lung recruitment strategy is still awaiting better answers.
